# The synergistic impact of NSAIDs and aggressive hydration therapy on the rate of post-ERCP pancreatitis in high -risk and low -risk patients 

**Published:** 2020

**Authors:** Morteza Aghajanpoor Pasha, Pegah Eslami, Arash Dooghaie Moghadam, Bobak Moazzami, Sajad shojaee, Faezeh Almasi, Narjes Tavakolikia, Mohsen Norouzinia, Ebrahim Radinnia, Amir Sadeghi

**Affiliations:** 1 *Gastroenterology and Hepatobiliary Research Center, AJA University of Medical Sciences, Tehran, Iran*; 2 *Gastroenterology and Liver Diseases Research Center, Research Institute for Gastroenterology and Liver Diseases, Shahid Beheshti University of Medical Sciences, Tehran, Iran*; 3 *Network of Immunity in Infection, Malignancy and Autoimmunity (NIIMA), Universal Scientific Education and Research Network (USERN), Tehran, Iran*; 4 *Basic and Molecular Epidemiology of Gastrointestinal Disorders Research Center, Research Institute for Gastroenterology and Liver Diseases, Shahid Beheshti University of Medical Sciences, Tehran, Iran*; 5 *Department of Internal Medicine, Loghman Hakim Hospital, Shahid Beheshti University of Medical Sciences, Tehran, Iran*; 6 *Social and Preventive Medicine Specialist, Head of Family, Population Health Department, Tehran University of Medical science, Tehran, Iran*

**Keywords:** Endoscopic retrograde cholangiopancreatography (ERCP), post ERCP pancreatitis

## Abstract

**Aim:**

The main complication of Endoscopic retrograde cholangiopancreatography (ERCP) is post-ERCP pancreatitis (PEP).

**Background:**

Based on demographic characteristics and underlying issues and ERCP indication, patients are categorized as high risk or low risk. There have been no studies on the synergistic effects of NSAIDS and hydration therapy, separately sorted by the risk assessment of PEP in different groups of patients.

**Methods:**

This study included 281 eligible participants after exclusion. According to demographic characteristics and co-morbidities, the patients were divided to high risk and low risk. The high-risk group was divided randomly into two subgroups and both of them received NSAIDs (100 mg rectal Diclofenac). One group received standard hydration (1.5mg/kg/hr), another the other received aggressive hydration (3mg/kg/h). The low-risk group received standard hydration. One of its subgroups received NSAIDs, while others did not. The efficacy of these preventions was compared across 4 subgroups.

**Results:**

The mean age was 59.85±17.17. Eight hours after ERCP, the amylase and lipase were significantly higher in the high-risk group with standard hydration (P=0.00). Amylase, lipase 8 hours, between two low risk subgroups, NSAIDs had no significant effect (P=0.38, P=0.95, respectively). After adjustment based on cannulation, manipulation and duration of time, the results had no change (P=0.64, P=0.19, P=0.61).

**Conclusion:**

The aggressive hydration could significantly decrease the risk of PEP. However, the low-risk group was exposed to the lowest risk of PEP. NSAIDs could not help to decrease the rate PEP in the low-risk groups alone. Overall, it seems hydration and NSAIDs therapy had synergistic outcome in high-risk patients.

## Introduction

 Endoscopic retrograde cholangiopancreatography (ERCP) is a powerful tool that allows fluoroscopic and endoscopic access to a pancreaticobiliary tree for gastroenterologists, for approximately more than five decades (1). Post-ERCP pancreatitis (PEP) from those decades remains the most frequent complication such that in the United States, it costs more than $ 150 million annually (2). According to previous reports, PEP has occurred in 2-10% of patients undergoing ERCP; however, in a high-risk group, it ranges between 8-40% (2). Also, the mortality rate of PEP is estimated at nearly 0.7% (3). Both patient and technique-related risk factors are associated with a higher incidence of PEP. Patient-related risk factors include age of less than 60 years, history of PEP, normal serum bilirubin levels, and dysfunction of Oddi Sphincter. Moreover, technique-related risk factors include precut and trauma during sphincterotomy, frequent intrapancreatic duct contrast injection, balloon dilatation, and difficult cannulation (4).

Nowadays, pharmacological and mechanical prophylaxis such as pancreatic duct stenting and using rectal nonsteroidal anti-inflammatory drugs (NSAIDs) are used for preventing PEP (5). Rectal usage of NSAIDs and pancreatic duct stenting has shown a 40% and 60% reduction in PEP incidence, respectively (6, 7). Due to this reduction, rectal NSAIDs are routinely used before ERCP for all patients based on the recommendations of The European Society for Gastrointestinal Endoscopy (ESGE) for the high-risk group (8). Periprocedural hydration is another choice for reducing PEP by the mechanism of saving sufficient tissue perfusion during ERCP (9). Recently, a cohort study has demonstrated the inverse relationship between periprocedural hydration and the severity of PEP (10). In another study, aggressive hydration protocol showed a better preventive effect in comparison with normal hydration protocol (11, 12). Moreover, NSAIDs and hydration have synergism effect on PEP because of different mechanism . Thus, they suppress inflammation and stabilize tissue circulation during the procedure (9). A randomized double-blinded study showed that lactated Ringer’s Solution with Rectal Indomethacin reduced PEP in the high-risk patient (13).

To date, several trials have been designed to prevent PEP. Nevertheless, the majority of these trials have analyzed hydration with lactated ringer solutions or other choices of prevention separately against PEP in a high-risk group while few studies have focused on a combination therapy in high-risk groups. In the present study, we attempted to compare the effectiveness of aggressive hydration plus rectal NSAIDs protocol with standard hydration plus rectal NSAIDs in high-risk groups. Moreover, we compared the obtained results with the results of only normal hydration receiver and normal hydration plus NSAIDs in normal patients to propose the best strategy for reducing PEP and its burden on the health system. 

## Methods

**Patients**

In this single-center randomized clinical trial, the participants were comprised of 281 patients, who referred to our endoscopy unit in Taleghani Hospital as a territory referral ERCP center, and underwent ERCP according to routine indications from February 2014 to September 2017. The patients aged below 70 years were included in this study, and the exclusion criteria were as follows: (1) hyponatremia, (serum Na^+^ levels < 130 ), (2) hypernatremia (serum Na^+^  > 150 mmol/L), (3) renal insufficiency, (4) pulmonary edema (pO_2_ < 60 millimetre of mercury or saturation < 90% despite FiO_2_ of 30% or needing mechanical ventilation), (5) hepatic failure (cirrhosis and ascites), (6) NYHA cardiac function status ii and above, (7) pregnancy, (8) u-stent insertion, , (9) history of GI bleeding and other complications caused by NSAIDS, and (10) hypotension (SBP< 90 mmHg or MAP < 70 mmHg).

With regard to the inclusion and exclusion criteria, 320 patients participated in this study. The participants were first assessed in terms of the following criteria and were then assigned to high-risk and low-risk groups. In this study, a high-risk patient is the one meeting one (or more) of these criteria: 

1) age below 60 years old; 2) Oddi sphincter dysfunction; 3) normal bilirubin level; 4) positive history of pancreatitis; 5) cannulation duration time more than 10 minutes; 6) a repeated cannulation; 7) different technique trying for cannulation; 8) biliary dilation without sphincterotomy; and 9) injection of contrast in pancreatic duct

**Study design**

The high-risk patients were also randomly assigned to standard and aggressive hydration therapy groups, both of whom received rectal nonsteroidal anti-inflammatory drugs (NSAIDs). In another group, the low-risk patients were randomly divided to standard hydration and standard hydration plus rectal NSAIDS group after the finalization of therapeutic or diagnostic ERCP. A simple randomization technique was adopted for randomization with no stratification factor. After obtaining therapeutic or diagnostic ERCP, the suitability of this technique was confirmed, and the risk assessment was performed by a physician. Accordingly, the patients were divided into high-risk and low-risk groups. The study assistant assigned the patients to either NSAIDs plus aggressive hydration /NSAIDs plus standard hydration high-risk groups or standard hydration/standard hydration plus NSAIDs low-risk groups using a uniform random number algorithm. The written informed consent forms were submitted to all the patients before the study. This study was approved by the ethics committee of Medical Sciences of Shahid Beheshti University. The project was conducted in accordance with Declaration of Helsinki and the related protocols.

**Endoscopic procedures and patient cares**

All ERCP in our studies was performed under the supervision of a consultant having the experience of performing 1000 ERCP procedure. In this study, Fujinon ED 450x4 duodenoscope was used. First, our team collected the patients’ demographic data and past medical history. Afterwards, before performing ERCP, the patients were assessed once more by our medical team, and further physical examinations were performed. Prior to the concerned procedure, the patients underwent general anesthesia, and their vital signs were monitored during this procedure. After dividing the patients into high-risk and low-risk groups, the high-risk patients received 100 mg diclofenac sodium rectally 30 minutes before the procedure, and a group of these patients then randomly received lactate ringer solution 1.5 ml/kg/h during ERCP. With the completion of ERCP procedure, the patients received the same dosage of lactate ringer solution for about 8 hours. The high-risk group received lactate ringer as aggressive regimen, 3 ml/kg/h during ERCP, and additional 20 ml/kg/h bolus dose of this solution were infused at the end of the procedure, followed by 8 hours infusion of 3 ml/kg/h lactate ringer solution.

In the low-risk group, all the patients first received 1.5 ml/kg/h of lactate serum during the procedure and then 1.5 ml/kg/h of the same solution for 8 hours after the procedure. Furthermore, a group of the patients in the low-risk group randomly received 100 mg diclofenac sodium suppositories.

**Definitions**

In this study, post-ERCP pancreatitis is defined based on Cotton Criteria (14). Our institute specifies post-ERCP pancreatitis as a persistent epigastric or periumbilical pain along with hyperamylasemia, which is represented amylase enzyme amounts 3 times more than normal upper limits. In addition, we defined mild pancreatitis as the nonexistence of systemic complication or organ failure; moderate pancreatitis as a condition with organ failure and the local or systemic complications settled within the first 48 hours after the procedure; and severe pancreatitis as a condition with organ failure and the local or systemic complications persisting even after 48 hours after ERCP.

**Table 1:** The frequency and prevalence of high-risk subgroups with standard hydration and aggressive hydration, and low-risk subgroups with received NSAID, or absence of it

Group	Percent (%)	Frequency
High risk 1	21.7	61
High risk 2	21.4	60
Low risk 1	30.6	86
Low risk 2	26.3	74

**Table 2:** The prevalence and frequency of patients according to manipulation time, duration of procedure, and cannulation method

Adjusted factors	Subgroups of factors	Percent (%)	Frequency
Manipulation time	<5 min 5-10 min >10 min	76.9 14.9 8.2	216 42 23
Duration of procedure	<20 min ≥20 min	54.8 45.2	154 127
Cannulation method	Standard sphinctrotomy Precut sphinctrotomy Precut fistulotomy others	70.5 18.5 6.8 3.2	198 55 19 9

**Table 3:** The statistical analysis, the comparison of levels of the first hour and 8^th^ hours amylase an lipase between high-risk and low-risk subgroups with different hydration and NSAIDs

Enzyme ( between groups)	F	P- value
Amylase 1	0.73	0.53
Amylase 2	6.62	0.00
Lipase 1	1.07	0.36
Lipase 2	6.46	0.00

**Statistical analysis**

Categorical data were described as frequencies and percentages. Dependent variables including Amylases and Lipases Enzymes were compared by one-way ANOVA in high- and low-risk groups. Also, Tukey’s HSD Post-hoc analysis was conducted for multiple comparisons across groups. Randomized complete block design (RCBD) with replication was applied for investigating the significance of Enzymes by adjusting cannulation, duration and manipulation. All statistical analyses were performed by SPSS version 23.0. The level of significance was set at p<0.05 in all analyses 

## Results

After exclusion, 281 eligible participants were selected in our study. According to the criteria for determining patients with low-risk pancreatitis, 160 patients were allocated to the high-risk group, and 121 patients were assigned to the low-risk group (table 1).

Nearly, 62% of all participants were female. The mean age of our study population was 59.85±17.17 years old. CBD stone or its dilation was the main cause of ERCP procedure (88%). Furthermore, in more than 95% of these participants, ERCP was afforded successfully. The manipulation time continued less than 5 minutes in 76.9% of participants. The duration of procedure in 54.8% was less than 20 minutes. Sphinctrotomy was the most common method of cannulation among our patients (70.5%). In less than 40% of patients, stent was placed/contrived. Finally, pancreatitis occurred in less than 4% of patients in both groups (table 2.)

One hour after the procedure, Amylase and lipase levels as the predictive factors of pancreatitis showed no significant difference among these four groups (p=0.53, p=0.36 respectively). However, the amylase and lipase levels revealed significant changes between these groups (P=0.00, P=0.00) eight hours after the procedure. In this case, the high-risk subgroup 1 had significantly higher levels of amylase and lipase in the same period in comparison to other subgroups (574.11, 485, 97 respectively). Between the 2 subgroups of low-risk patients, no no significant difference was detected for the same procedure and level (P=0.38, P=0.95) (table 3.)

In this study, all the results were adjusted according to manipulation time, duration of procedure and cannulation method. 

**Adjusted results based on manipulation time**

Based on the manipulation time, three times were described, including manipulation time of less than 5 minutes, between 5-10 minutes and more than 10 minutes. After the adjustment, the results did not change according to the manipulation time. Manipulation had no significant effect on changing the results (P=0.196)

**Adjusted results based on time duration**

Furthermore, all the results were estimated after adjustment based on the duration of ERCP procedure. Results did not alter after the adjustment (P=0.61)

**Adjusted results based on cannulation method**

Finally, the results were adjusted according to cannulation methods. The methods in this study included standard sphinctrotomy, precut sphinctrotomy and precut fistulotomy. The adjustment could not significantly alter the main results based on the type of cannulation in our study (P=0.64).

## Discussion

ERCP procedure could reverse the flow of bile into the pancreas, which together with contrast injection would worsen inflammation in pancreas and cause post-ERCP acute pancreatitis (12). We assigned the patients to high-risk and low-risk groups. In our study, more than 50% of the participants were female, and the rate of pancreatitis was higher among the female patients.

Amylase level is one of the main and early predictor enzymes to detect PEP at the first convenient time. Amylase level in post-ERCP acute pancreatitis usually rises from one hour after the procedure and returns to a normal range approximately after 48 hours (16). According to previous evidence, the optimal time with the highest sensitivity for the detection of PEP based on the amylase level is eight hours after the procedure (17). Therefore, we designed a 1-hour amylase measurement as the initiation time of rising amylase in PEP and an eight-hour amylase measurement as the optimal time of increasing this enzyme. In this regard, the lipase serum level seems to better detect PEP during similar durations, in comparison to the amylase level; hence, the present study measured lipase and amylase simultaneously for the better detection of PEP cases.

An optimal hydration therapy during the first day of ERCP procedure could decrease the cytokines causing acute pancreatitis (18). Pancreatitis could decrease the incidence and severity of acute pancreatitis at the same time (18). In a meta-analysis, Zhang et al. evaluated seven RCT cases to examine the effects of hydration on the rate of acute pancreatitis among patients who underwent ERCP (12). This study showed that the aggressive hydration with at least 3900 ml ringer lactate during 9 hours could effectively prevent PEP. However, the studies included in this meta-analysis did not determine the role of NSAIDs in terms of aggressive hydration (12). Furthermore, they analyzed the effect of aggressive hydration therapy in a non-selective sample as they did not separate the patients by PEP risk. On the other hand, most of these studies focused on the amylase level to examine PEP (12). Another similar meta-analysis in 2019 reported that aggressive hydration could prevent PEP and reduce the hyperamylasemia in patients (19). Jun-Ho Choi’s study focused on a high-risk population and revealed that the hydration with ringer lactate pre-ERCP could decrease PEP risk in the high- and average-risk groups. The aggressive hydration in their study was described as at least 3mg/kg/h hydration before the procedure (20). In a review study by Hamada, he suggested that future studies should focus on the role of NSAIDs and compare the findings for the high-risk and low-risk subgroups (21). Accordingly, we decided to include four subgroups of low-risk and high-risk arms to evaluate the role of NSAIDs and aggressive hydration. The high-risk group, in comparison to another high risk group, received standard hydration, and the same NSAID provided significantly better outcomes. This finding, however, revealed that the aggressive hydration plus NSAIDs could reduce PEP risk in the high-risk group, but it was not as safe as the low-risk groups. Accordingly, the reduction of the risk factors of PEP should be of concern. Several studies examined the role of NSAIDs, oral or rectal, in decreasing the incidence of PEP. Accordingly, the one or two dosage of NSAIDs, before the start of the procedure could not increase the rate of GI bleedings due to NSAIDs (22). Therefore, it could be effective with limited side effects. However, most of these studies evaluated the effect of NSAIDs in high-risk groups, and the role of these agents in decreasing PEP in low-risk patients has been less addressed. In addition, these studies have rarely investigated the role of synergetic effects of NSAIDs and aggressive hydration on the incidence of PEP in the high-risk patients (22). Previous studies estimated the incidence rate of PEP to approximately 3-4% in patients who received rectal indomethacin. This estimation agrees with the findings of the present study. In our study, both high-risk subgroups and one of the low-risk arms were administrated 100 mg rectal Diclofenac before the procedure. Among the PEP patients participated in our study, only one patient presented the severe type. Furthermore, based on previous studies, NSAIDs seem to be effective in reducing the duration and severity of PEP as well (22). Hosseini et al. reported that pre-ERCP indomethacin prescription and hydration simultaneously not only reduced the incidence rate of PEP (4%) in comparison to the control group (17-19%) but it also could decrease the severity of such a complication. Furthermore, they that the side effects of this prevention agent are similar in the case and control groups (22). However, the findings of the studies conducted in this regard are inconsistent. Lourdes del-Olmo-Martinez et al. reported that application of the pre-ERCP diclofenac had no protective effect against PEP in patients who were not selected based on high or low levels of risk (23). A meta-analysis conducted in 2016 on six studies and 2473 patients revealed that rectal indomethacin did not significantly reduce PEP rate (24). In our study, PEP rate was similar to that reported in Hosseini’s study. In contrast, the patients in our study were categorized as the high-risk and low-risk patients, and the roles of hydration and NSAIDs prevention therapy in these two groups of participants were compared. Our findings showed that, with the exception of disruptive factors in the low-risk group, 100 mg diclofenac caused no significant difference between the two subgroups receiving the same hydration therapy. Considering the disruptive factors such as manipulation time and overall duration of procedure, it can be seen that 100 mg diclofenac causes no significant differences between the two subgroups of low-risk patients. In general, however, it could help to decrease PEP risk in high-risk and low-risk patients. Our study, in comparison to the previous studies, revealed that this prevention agent had significant outcomes only in the high-risk group (23, 25). However, other studies suggested that indomethacin could have better effects on patients with moderate PEP risk (25). A large body of research has dealt with the optimal dosage and the form of usage. In their case control study, Uçar et al. compared 75 mg diclofenac intramuscular with 100 mg rectal diclofenac and showed that 100mg rectal diclofenac had significantly better effects on decreasing PEP (26) (27, 28). Therefore, we decided to prescribe 100mg rectal diclofenac in our study design for an optimal achievement.

In the present study, several factors, which could have effects on PEP, were adjusted and their effects on the final incidence of PEP were evaluated in four different groups. These factors included manipulation time, duration of procedure, and cannulation method.

Most of our participants were candidates for the standard sphinctrotomy. In difficult cannulation cases, the precut techniques were suggested. In a few participants, NKFs were preferred. According to the previous studies, although the precut cannulation methods seem to increase the number of successful cases, these techniques are accompanied with more serious complications, especially post-ERCP pancreatitis (29). In contrast, in a meta-analysis by Gong et al., there was no significant increase in the number of successful cannulation using precut techniques, and the simple form of precut techniques could decrease the incidence rate of PEP (30, 31). A new epidemiological study on 1786 cases reported no significant role for cannulation method in rising the incidence rate of PEP (15). In general, there is no agreement among the findings existing in the literature. In our study, we adjusted our results to the type of cannulation method to achieve more accurate outcomes. The adjusted result showed that cannulation method had no significant effect on the results, and we could not consider it as a disruptive parameter. Furthermore, the same finding was achieved regarding the role of manipulation and duration time. 

Our study has some limitations. First, it was a single-centre retrospective study. Moreover, we compared neither the effects of NSAIDS among the high-risk subgroups nor the effects of the aggressive therapy on the low-risk subgroups. 

This is a novel study on comparing the role of aggressive therapy and NSAIDs at same time among the low-risk and high-risk patients. Notably, the aggressive hydration along with NSAIDs could significantly provide better outcomes among high-risk patients. On the other hand, NSAIDs had no effect on the low-risk patients. To sum up, future studies are recommended to separate the low-risk and high-risk patients in order to confirm the present findings and set a detailed protocol for preventing PEP among high-risk and low-risk patients.

## Conflict of interests

The authors declare that they have no conflict of interest.

## References

[1] McCune WS, Shorb PE, Moscovitz H (1968). Endoscopic cannulation of the ampulla of vater: a preliminary report. Ann Surg.

[2] Committee ASoP, Anderson MA, Fisher L, Jain R, Evans JA, Appalaneni V (2012). Complications of ERCP. Gastrointest Endosc.

[3] Kochar B, Akshintala VS, Afghani E, Elmunzer BJ, Kim KJ, Lennon AM (2015). Incidence, severity, and mortality of post-ERCP pancreatitis: a systematic review by using randomized, controlled trials. Gastrointest Endosc.

[4] Wu D, Wan J, Xia L, Chen J, Zhu Y, Lu N (2017). The Efficiency of Aggressive Hydration With Lactated Ringer Solution for the Prevention of Post-ERCP Pancreatitis: A Systematic Review and Meta-analysis. J Clin Gastroenterol.

[5] Sotoudehmanesh R, Ali-Asgari A, Khatibian M, Mohamadnejad M, Merat S, Sadeghi A (2019). Pharmacological prophylaxis versus pancreatic duct stenting plus pharmacological prophylaxis for prevention of post-ERCP pancreatitis in high risk patients: a randomized trial. Endoscopy.

[6] Choudhary A, Bechtold ML, Arif M, Szary NM, Puli SR, Othman MO (2011). Pancreatic stents for prophylaxis against post-ERCP pancreatitis: a meta-analysis and systematic review. Gastrointest Endosc.

[7] Patai Á, Solymosi N, Mohácsi L, Patai Á V (2017). Indomethacin and diclofenac in the prevention of post-ERCP pancreatitis: a systematic review and meta-analysis of prospective controlled trials. Gastrointest Endosc.

[8] Dumonceau JM, Andriulli A, Elmunzer BJ, Mariani A, Meister T, Deviere J (2014). Prophylaxis of post-ERCP pancreatitis: European Society of Gastrointestinal Endoscopy (ESGE) Guideline - updated June 2014. Endoscopy.

[9] Smeets XJNM, da Costa DW, Fockens P, Mulder CJJ, Timmer R, Kievit W (2018). Fluid hydration to prevent post-ERCP pancreatitis in average- to high-risk patients receiving prophylactic rectal NSAIDs (FLUYT trial): study protocol for a randomized controlled trial. Trials.

[10] Sagi SV, Schmidt S, Fogel E, Lehman GA, McHenry L, Sherman S (2014). Association of greater intravenous volume infusion with shorter hospitalization for patients with post-ERCP pancreatitis. J Gastroenterol Hepatol.

[11] Shaygan-Nejad A, Masjedizadeh AR, Ghavidel A, Ghojazadeh M, Khoshbaten M (2015). Aggressive hydration with Lactated Ringer's solution as the prophylactic intervention for postendoscopic retrograde cholangiopancreatography pancreatitis: A randomized controlled double-blind clinical trial. J Res Med Sci.

[12] Zhang ZF, Duan ZJ, Wang LX, Zhao G, Deng WG (2017). Aggressive Hydration With Lactated Ringer Solution in Prevention of Postendoscopic Retrograde Cholangiopancreatography Pancreatitis: A Meta-analysis of Randomized Controlled Trials. J Clin Gastroenterol.

[13] Mok SRS, Ho HC, Shah P, Patel M, Gaughan JP, Elfant AB (2017). Lactated Ringer's solution in combination with rectal indomethacin for prevention of post-ERCP pancreatitis and readmission: a prospective randomized, double-blinded, placebo-controlled trial. Gastrointest Endosc.

[14] Williams EJ, Taylor S, Fairclough P, Hamlyn A, Logan RF, Martin D (2007). Risk factors for complication following ERCP; results of a large-scale, prospective multicenter study. Endoscopy.

[15] Li G-Z, Wang F, Fang J, Zha H-L, Zhao Q (2018). Risk Factors for Post-Endoscopic Retrograde Cholangiopancreatography Pancreatitis: Evidence from 1786 Cases. Med Sci Monit.

[16] Lee YK, Yang MJ, Kim SS, Noh CK, Cho HJ, Lim SG (2017). Prediction of Post-Endoscopic Retrograde Cholangiopancreatography Pancreatitis Using 4-Hour Post-Endoscopic Retrograde Cholangiopancreatography Serum Amylase and Lipase Levels. J Korean Med Sci.

[17] Testoni PA, Caporuscio S, Bagnolo F, Lella F (1999). Twenty-four-hour serum amylase predicting pancreatic reaction after endoscopic sphincterotomy. Endoscopy.

[18] Coté GA (2014). Intravenous Hydration for the Prevention of Post-Endoscopic Retrograde Cholangiopancreatography Pancreatitis. Gastroenterology.

[19] Radadiya D, Devani K, Arora S, Charilaou P, Brahmbhatt B, Young M (2019). Peri-Procedural Aggressive Hydration for Post Endoscopic Retrograde Cholangiopancreatography (ERCP) Pancreatitis Prophylaxsis: Meta-analysis of Randomized Controlled Trials. Pancreatology.

[20] Choi JH, Kim HJ, Lee BU, Kim TH, Song IH (2017). Vigorous Periprocedural Hydration With Lactated Ringer's Solution Reduces the Risk of Pancreatitis After Retrograde Cholangiopancreatography in Hospitalized Patients. Clin Gastroenterol Hepatol.

[21] Hamada T, Nakai Y, Isayama H, Koike K (2017). Toward routine use of non-steroidal anti-inflammatory drugs for patients undergoing endoscopic retrograde cholangiopancreatography. Dig Endosc.

[22] Hosseini M, Shalchiantabrizi P, Yektaroudy K, Dadgarmoghaddam M, Salari M (2016). Prophylactic Effect of Rectal Indomethacin Administration, with and without Intravenous Hydration, on Development of Endoscopic Retrograde Cholangiopancreatography Pancreatitis Episodes: A Randomized Clinical Trial. Arch Iranian Med.

[23] Del Olmo Martínez L, Velayos Jiménez B, Almaraz Gómez A (2018). Rectal diclofenac does not prevent post-ERCP pancreatitis in consecutive high-risk and low-risk patients. Rev Esp Enferm Dig.

[24] Feng Y, Navaneethan U, Zhu X, Varadarajulu S, Schwartz I, Hawes R (2017). Prophylactic rectal indomethacin may be ineffective for preventing post-endoscopic retrograde cholangiopancreatography pancreatitis in general patients: A meta-analysis. Dig Endosc.

[25] Lyu Y, Cheng Y, Wang B, Xu Y, Du W (2018). What is impact of nonsteroidal anti-inflammatory drugs in the prevention of post-endoscopic retrograde cholangiopancreatography pancreatitis: a meta-analysis of randomized controlled trials. BMC Gastroenterol.

[26] Uçar R, Biyik M, Uçar E, Polat İ, Çifçi S, Ataseven H (2016). Rectal or intramuscular diclofenac reduces the incidence of pancreatitis after endoscopic retrograde cholangiopancreatography. Turk J Med Sci.

[27] Khoshbaten M, Khorram H, Madad L, Ehsani Ardakani MJ, Farzin H, Zali MR (2008). Role of diclofenac in reducing post-endoscopic retrograde cholangiopancreatography pancreatitis. J Gastroenterol Hepatol.

[28] Tryliskyy Y, Bryce GJ (2018). Post-ERCP pancreatitis: Pathophysiology, early identification and risk stratification. Adv Clin Exp Med.

[29] de la Morena Madrigal EJ, Rodríguez García Mª I, Galera Ródenas AB, Pérez Arellano E (2018). Biliary cannulation effectiveness and pancreatitis risk using two early precut techniques. Rev Esp Enferm Dig.

[30] Gong B, Hao L, Bie L, Sun B, Wang M (2010). Does precut technique improve selective bile duct cannulation or increase post-ERCP pancreatitis rate? A meta-analysis of randomized controlled trials. Surg Endosc.

[31] Katsinelos P, Gkagkalis S, Chatzimavroudis G, Beltsis A, Terzoudis S, Zavos C (2012). Comparison of three types of precut technique to achieve common bile duct cannulation: a retrospective analysis of 274 cases. Dig Dis Sci.

